# Analysis of clinical failure of anti-tau and anti-synuclein antibodies in neurodegeneration using a quantitative systems pharmacology model

**DOI:** 10.1038/s41598-023-41382-0

**Published:** 2023-09-01

**Authors:** Hugo Geerts, Silke Bergeler, Mike Walker, Piet H. van der Graaf, Jean-Philippe Courade

**Affiliations:** 1 Certara US, 100 Overlook Centre, Suite 101, Princeton, NJ 08540 USA; 2 Certara UK, Canterbury Innovation Centre, University Road, Canterbury, CT2 7FG Kent UK; 3 discoveric bio alpha, Bahnhofstrasse 1, 8808 Pfäffikon, Switzerland; 4 Present Address: Bristol-Meyers-Squibb, Lawrenceville, NJ 08648 USA

**Keywords:** Drug discovery, Neuroscience, Neurology

## Abstract

Misfolded proteins in Alzheimer’s disease and Parkinson’s disease follow a well-defined connectomics-based spatial progression. Several anti-tau and anti-alpha synuclein (aSyn) antibodies have failed to provide clinical benefit in clinical trials despite substantial target engagement in the experimentally accessible cerebrospinal fluid (CSF). The proposed mechanism of action is reducing neuronal uptake of oligomeric protein from the synaptic cleft. We built a quantitative systems pharmacology (QSP) model to quantitatively simulate intrasynaptic secretion, diffusion and antibody capture in the synaptic cleft, postsynaptic membrane binding and internalization of monomeric and oligomeric tau and aSyn proteins. Integration with a physiologically based pharmacokinetic (PBPK) model allowed us to simulate clinical trials of anti-tau antibodies gosuranemab, tilavonemab, semorinemab, and anti-aSyn antibodies cinpanemab and prasineuzumab. Maximal target engagement for monomeric tau was simulated as 45% (semorinemab) to 99% (gosuranemab) in CSF, 30% to 99% in ISF but only 1% to 3% in the synaptic cleft, leading to a reduction of less than 1% in uptake of oligomeric tau. Simulations for prasineuzumab and cinpanemab suggest target engagement of free monomeric aSyn of only 6–8% in CSF, 4–6% and 1–2% in the ISF and synaptic cleft, while maximal target engagement of aggregated aSyn was predicted to reach 99% and 80% in the synaptic cleft with similar effects on neuronal uptake. The study generates optimal values of selectivity, sensitivity and PK profiles for antibodies. The study identifies a gradient of decreasing target engagement from CSF to the synaptic cleft as a key driver of efficacy, quantitatively identifies various improvements for drug design and emphasizes the need for QSP modelling to support the development of tau and aSyn antibodies.

## Introduction

Interest in Tau immunotherapy in the Alzheimer’s disease (AD) field has increased due to clinical observations that tau pathology has a great impact on clinical progression^1^ and that spatial progression of tau pathology can be observed both in preclinical models^2^ and in the human brain^3^. Preclinical evidence exists that aSyn can also spread from cell to cell, opening the field to immunotherapy to capture these extracellular species, following the example of beta-amyloid antibodies and the recent success of lecanemab. However, while beta-amyloid oligomers and plaques mostly reside in the extracellular space with easier antibody access, misfolded tau and aSyn are mostly intracellular proteins. Only a small portion is excreted into the extracellular space with antibody access^2, 4, 5^.

The first clinical trials for both anti-tau and anti-aSyn antibodies have been disappointing. Despite substantial target engagement (> 95% at the highest dose) in CSF samples in Phase 1 studies for gosuranemab^6^, the drug failed to change clinical outcomes or relevant imaging biomarkers in longer Phase 2 trials in Progressive Supranuclear Palsy (PSP)^7^. Both tilavonemab in progressive supranuclear palsy (PSP)^8^ and semorinemab in AD^9^ failed to change clinical progression. In Parkinson’s disease (PD), cinpanemab^10^ and prazinuezumab^11^ were also inactive in longer Phase 2 trials. Several suggestions have been proposed to explain the discrepancies between the pharmacodynamic CSF readouts and clinical outcomes. An important consideration could be the mismatch between the antibody epitope and the oligomeric part of the protein^12, 13^. Indeed, often recombinant forms (tau) or sonicated preformed fibril a-synuclein (PFF) are used to generate the therapeutic antibodies, however it is worth noting that the complexity of human brain seeds (multiple isoforms, multiple post translational modifications, truncations) is not fully recapitulated. We will use the term “oligomers” for all forms of misfolded tau or Asyn proteins.

While it took well over 15 years for beta amyloid-based therapy to identify the right conditions for generating clinical success with lecanemab, we should attempt to accelerate the development of anti-tau and anti-aSyn therapies.

One approach is to learn from past trials and identify the possible reasons for failure to support better future clinical trial designs. This refers to both drug properties such as pharmacology and pharmacokinetics as well as patient intrinsic factors and the identification of relevant biomarkers reporting on the impact of the therapies at the site of action, as suggested in a recent article^14^. Unfortunately, current preclinical models or in vitro cultures of neuronally differentiated human iPSC cells or organoids can only recapitulate parts of human pathology and physiology.

In this report, we wanted to explore what other factors beyond epitope mismatch would drive the therapeutic response^14^. We used a computer-based modelling platform combining the knowledge of these different experimental approaches and human brain anatomical properties. A similar quantitative systems pharmacology (QSP) approach has been successfully applied to beta-amyloid therapies for beta-amyloid biomarkers^15–17^ and anticipated effects on functional clinical scales^18, 19^.

Our mechanism-based QSP model of tau and aSyn progression includes secretion of monomeric and oligomeric protein from presynaptic nerve endings, diffusion in the synaptic cleft, capture by antibodies, binding to heparan sulphate proteoglycan (HSPG,^20^ and LRP1^21^ on the postsynaptic membrane, uptake by pinocytosis (for tau)^22^, internalization and subsequent intraneuronal degradation. The model parameters were fit to align with experimentally determined uptake parameters in neuronal cultures^22–24^ and clinically determined tau/aSyn levels in CSF and brain parenchyma^25, 26^.

A PBPK model describing the distribution of anti-tau and anti-aSyn antibodies in plasma, CSF, ISF and synaptic cleft after i.v. injection in human subjects was built using a previously published model^27, 28^. It was based on the reported PK profiles, pharmacology dosing strategies and clinical CSF target engagement of gosuranemab^6^, tilavonemab^29^, and semorinemab^9^ for tau antibodies and cinpanemab^30^ and prasineuzumab^31^ for aSyn antibodies.

The platform identified possible reasons, both drug-related and drug-independent, for the discrepancy between the effect on CSF biomarkers and the uptake of oligomeric protein, which we assume to be a proxy for clinical progression. Even for those cases where the right epitope would be selected, we believe drug-independent insights from the QSP model could support better clinical trials.

## Methods

### PBPK model description

We use a simplified PBPK model derived from^27, 28^, focused on the brain distribution of biologics and constrained by experimental data in mice, rats, monkeys and humans, which itself expands upon the original PBPK model for antibody distribution over other tissues in the human body^32^. Each tissue is modelled with a vascular space, an endosomal space and an interstitial space. The brain compartment includes a vascular space; two endosomal spaces, one representing the blood–brain barrier and the other the blood–CSF barrier; an interstitial space; and a series of four compartments representing the CSF in the lateral ventricle, the third-fourth ventricle, the cisterna magna and the subarachnoid space. Flows allow the movement of the drug between the interstitial space and the CSF compartments, and both the interstitial space and the subarachnoid space have fluid flows draining into the lymph. The simplified model^28^ used here lumps all the nonbrain tissues into one large tissue that has parameters that are a weighted average of the parameters for the individual tissues and collapses the four CSF compartments in the brain into a single CSF compartment.

The ISF compartment is further subdivided into a parenchyma compartment comprised of nonneuronal cells, a synaptic cleft and an intraneuronal compartment. Volumes of CSF, brain vasculature, endothelial cell layer and lymph are derived from^27^. We assume that 58% of the brain volume is taken up by neurons, hence a volume of 0.76 l for the neuronal compartment^33, 34^. ISF volume was supposed to be approximately 20% of total brain volume^35^ or 0.26 l^27^. For the calculation of the total synaptic cleft, we assume a synaptic cleft width of 40 nm with a pre- and postsynaptic surface of approximately 0.1–1 µm^2^^36^, resulting in a volume between 4 × 10^–18^ to 4 × 10^–17^ l per synapse. It is estimated that there are approximately 10^11^ neurons in the human brain^34, 37^, each on average with 1000 synapses^38^, resulting in an approximate total synaptic cleft volume of 0.001 l.

### QSP model of tau and aSyn propagation

The model structure is identical for tau and aSyn progression except for the pinocytosis mechanism, which is unique for tau spreading.

**Figure 1:**
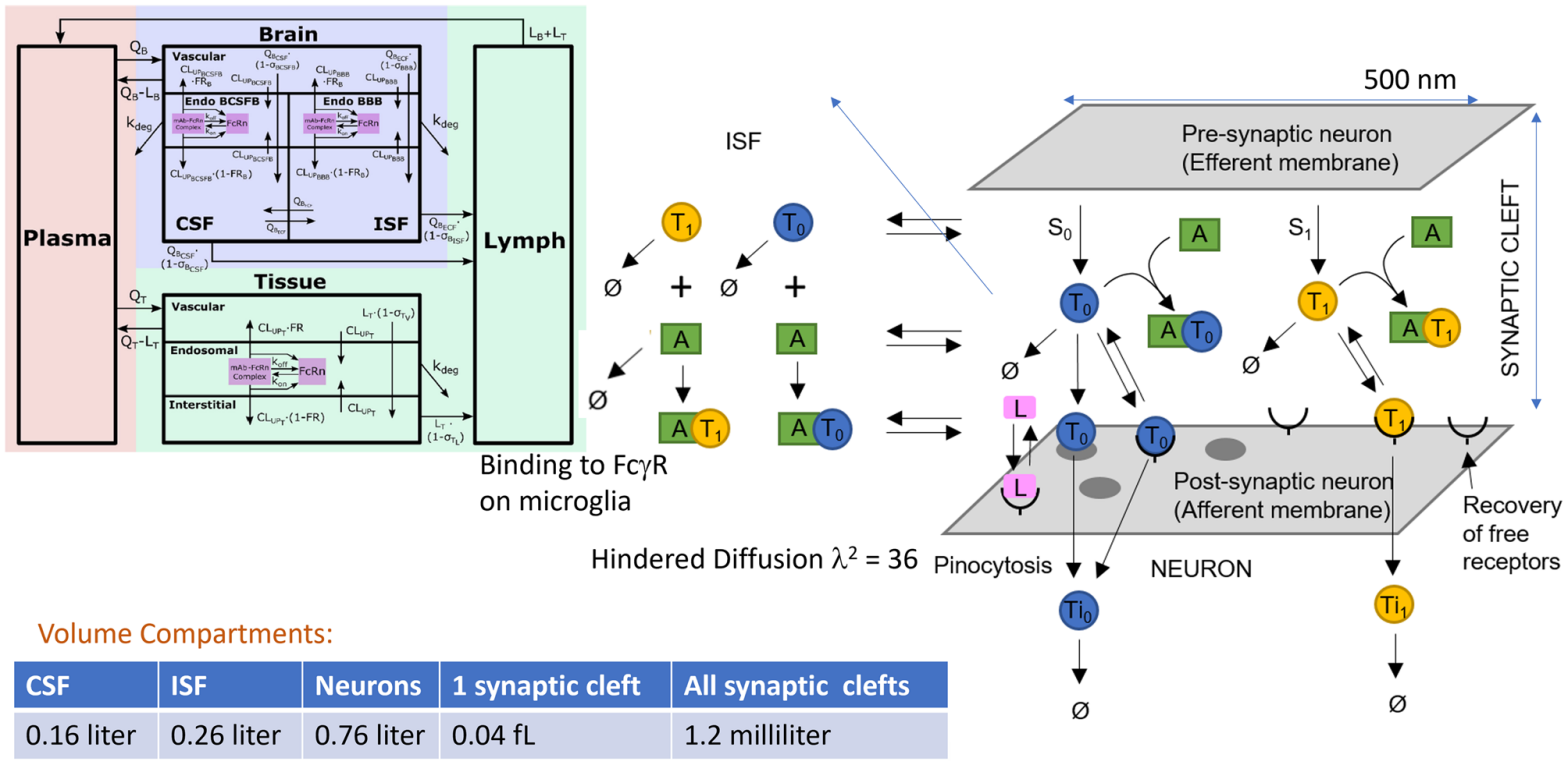
Schematic representation of the combined PBPK-QSP model. The model combines a minimal PBPK model (top left) with various brain compartments using appropriate volumes (bottom left) for derivation of both antibody concentrations in the various compartments (plasma, CSF, ISF, synaptic cleft) and levels of tau/aSyn in CSF. Availability of the antibodies is modulated by tortuosity and off-target capture by microglia in the ISF. The QSP part simulates key processes in the synaptic cleft**,** including secretion of free monomeric (blue) and oligomeric Tau/aSyn (yellow) from the presynaptic synaptic compartment, diffusion, binding to antibodies, binding to postsynaptic “acceptors (HSPG and LRP1)” and internalization of 2 forms of Tau/aSyn (monomeric and two oligomeric forms) into the postsynaptic neuronal compartment. This is assumed to be a proxy for misfolded protein propagation.

The mechanism-based QSP model of Tau/aSyn dynamics is illustrated in Fig. 1.

The QSP model describes the dynamics of different aSyn and tau species in the synaptic cleft, including secretion of monomeric and oligomeric forms, diffusion towards the postsynaptic membrane and out of the synapic cleft, interaction with antibodies dependent upon their exposure, binding to acceptors on the postsynaptic membrane, such as heparan sulfate proteoglycans (HSPG) and LRP1 in the case of tau, followed by internalization inside the postsynaptic neuronal compartment^39^.

The simulation is based on the following equation for each aSyn species:

1 $$\frac{d{T}_{i}}{dt}={S}_{i}-{f}_{i}^{R}{T}_{i}R+{b}_{i}^{R}{R}_{i}-{f}_{i}^{A}{T}_{i}A-kdeg* {T}_{i}$$

where i is a given Tau/aSyn species, T_i_ (t) is the time-dependent extracellular concentration of Tau/aSyn species i, S_i_ is the secretion rate of Tau/aSyn species i, f_i_^R^ is the forwards binding rate constant of Tau/aSyn species i to the receptor, R(t) is the concentration of unbound receptors, R_i_ (t) is the concentration of receptors bound by Tau/aSyn species i, b_i_ is the backward or unbinding rate constant of Tau/aSyn species i from the receptor, f_i_^A^ is the binding of Tau/aSyn species i to the antibody, A(t) is the free antibody concentration, and H_i_ is the half-life of Tau/aSyn species i (diffusion controlled).

The receptors bound by Tau/aSyn species i and those that are unbound are governed by the following ordinary differential equations (ODEs):

2 $$\frac{d{R}_{i}}{dt}={f}_{i}^{R}{T}_{i}R-\left({b}_{i}^{R}+{a}_{i}\right){R}_{i}$$

3 $$\frac{dR}{{dt}} = - \left( {\mathop \sum \limits_{i = 0}^{n - 1} f_{i}^{R} T_{i} } \right)R + \mathop \sum \limits_{i = 0}^{n - 1} b_{i}^{R} R_{i} + r\left[ {\left( {\overline{R} - \mathop \sum \limits_{i = 0}^{n - 1} R_{i} } \right) - R} \right]$$

where a_i_ is the absorption rate of Tau/aSyn species I, R-bar is the initial concentration of receptors and r is the recovery rate for bound receptors. Additionally, note that if $af{f}_{i}^{A}$ is the affinity of the antibody for Tau/aSyn species i (i.e., the ratio of unbinding to binding rates), we assume that once bound, it is highly unlikely for Tau/aSyn to unbind from the antibody, leading to the forwards rate constant ${f}_{i}^{A}=\frac{0.001}{af{f}_{i}^{A}}$ 1/(min*nM).

The amount of oligomeric Tau/aSyn internalized (or any Tau/aSyn species that enters the afferent neuron through HSPG binding) C_i_ (t) with kdeg the intraneuronal degradation rate for Tau/aSyn species is governed by the ODE

4 $$\frac{d{C}_{i}}{dt}={a}_{i}{R}_{i}-kdeg* {R}_{i}$$

For tau monomers, pinocytosis is an important process for tau internalization separate from receptor binding^22^. We assume that there is a concentration of ‘binding sites’, P(t), to which tau monomers can bind at a rate of ${f}_{0}^{P}$ to form *P*_*0*_ and then be internalized at the rate ${a}_{0}^{P}$, allowing more ‘binding sites’ to replace the ones that were absorbed, leading to the following two equations for tau monomers:

5 $$\frac{dP}{dt}= -{f}_{0}^{P}{T}_{o}P+{a}_{0}^{P}{P}_{0}$$

6 $$\frac{d{P}_{0}}{dt}= {f}_{0}^{P}{T}_{o}P-{a}_{0}^{P}{P}_{0}$$

Therefore, Eq. (7) governs the concentration C_0_ of tau internalized for monomers.

7 $$\frac{{\mathrm{dC}}_{0}}{\mathrm{dt}}={\mathrm{a}}_{0}{\mathrm{R}}_{0}+{\mathrm{a}}_{0}^{\mathrm{P}}{\mathrm{P}}_{0}-{k}_{deg}*{C}_{0}$$

## Results

### Calibration of tau and aSyn dynamics in longitudinal observational studies

Note that in all the following sections, tau0/aSyn0 refers to the monomeric form of tau/aSyn, and tau1/aSyn1 refers to the oligomeric or oligomeric tau/aSyn forms. Note that for the two anti-aSyn antibodies, “oligomeric” proteins are defined as preformed a-synuclein fibrils (PFFs).

A study on corneum cells^40^ suggests a glycan density between 106 and 1200 HSPG binding sites/µm^2^. A cell synapse has a surface area of approximately 1 µm^2^; therefore, a density of 10,000 nM/litre corresponds to 248 molecules. LRP1 (low-density lipoprotein receptor-related protein 1) also binds full-length nonphosphorylated tau with an affinity of 60 nM, but phosphorylated tau binds with a fourfold weaker affinity^21^. This constrains the parameters of tau/aSyn receptor density and affinity.

We first calibrated the uptake of fluorescent oligomeric tau into hiPSC cells differentiated into neurons^22^ and of tau purified brain extracts from AD and PSP patients into primary neuronal cultures^23^ (Figs. S1 and 2 and Table S1). The fraction of tau entering the neurons via pinocytosis is fitted to experimental data of fluorescent tau oligomer uptake in hiPSC neurons and to the number of puncta detected in the primary neuronal cell cultures, leading to the parameters for tau uptake (Table S1). The faster uptake of tau from PSP patients corresponded to a 120-fold higher affinity for the HSPG receptors.

Similarly, we calibrated the uptake of PFF aSyn into primary neuronal cultures^24^. Here, well-defined concentrations of fluorescently labelled PFF aSyn were applied to primary neuronal cultures, and uptake was measured after 1 h (Fig. S3). We assumed the level of oligomeric aSyn in all brain compartments to be between 25 and 30% of the free monomeric aSyn level, as measured in the CSF^41^.

Once we have calibrated the uptake parameters, we can then adjust the secretion rate to generate the experimentally determined steady-state levels of monomeric tau or aSyn in the CSF, resulting in average free CSF monomeric total tau levels of 180 pg/ml or 3 pM for healthy controls^42^. In AD patients, the level is approximately twice as high, resulting in a 6.7 pM CSF tau concentration. For PSP patients, we adjusted the secretion level to reflect the lack of increased CSF tau levels^43^.

Free monomeric ISF tau has been measured using microdialysis in traumatic brain injury (TBI) subjects^25^ and ranges from 200 to 4000 pg/ml or 3.3 to 66 pM. The resulting ISF free monomeric level calculated from the PBPK model of 8 pM for the ISF is in that range. Based on the identification of the small subset of total tau in Tg4510 Tg mice that is oligomeric^44^, we assume a ratio of 100:1 for monomeric over oligomeric protein.

The levels of free monomeric aSyn in longitudinal studies without intervention result in average free CSF monomeric aSyn levels of 1950 pg/ml or 112 pM for healthy controls^45, 46^.

Free monomeric ISF aSyn has also been measured using microdialysis in TBI subjects and ranges from 0.6 ng/ml (44 pM) to 9 ng/ml (620 pM)^26^. The ISF free monomeric level calculated from the PBPK model of 312 pM for the ISF is right in that range.

### Phase 1 single-dose trial modelling

**Figure 2:**
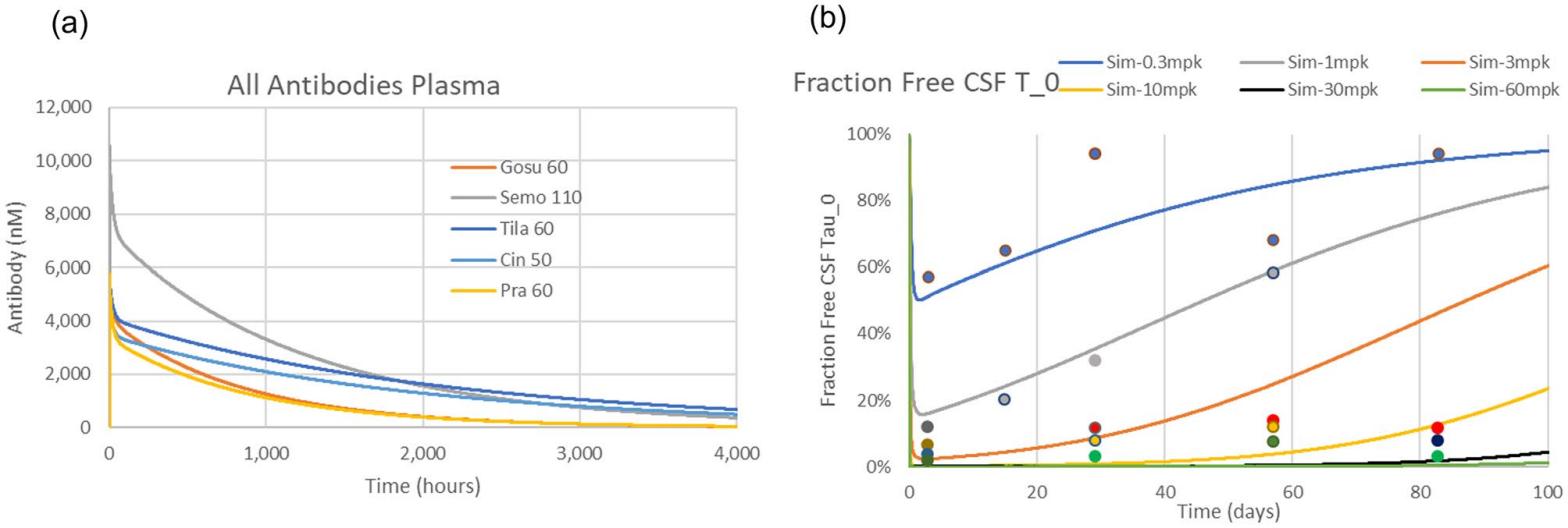
Plasma PK and pharmacodynamic effect. (**a**) Plasma PK profiles of the five antibodies at their highest dose from the fitted profiles in the Phase 1 study. (**b**) Simulated and experimentally observed pharmacodynamic effect of different gosuranemab doses on free CSF monomeric tau, normalized to baseline, as reported in their Phase 1 study (dots are experimentally reported data).

We first fitted reported plasma profiles of different anti-tau and anti-aSyn antibodies using the PBPK model (Figs. S4–S8 and Table S2). The PBPK model estimates the CSF and ISF concentrations of the antibodies with CSF concentrations ranging between 0.1 and 0.4% of plasma exposures and ISF levels approximately 70% lower (0.07–0.3% of plasma exposures). It is of interest to note the different half-life of the five antibodies, as this will determine the accumulated effect on neuronal uptake of oligomeric protein (Fig. 2). A vastly different PK profile and half-life were observed. Tilavonemab had the longest half-life (650–880 h), 2.5- to 3.4-fold higher than prasineuzumab, which had the shortest half-life (260 h) (Table 1). Surprisingly, despite the differences in half-life and affinity, the clinical doses used were similar (30–60 mg/kg). The parameters for the PBPK model are listed in Table S2. Based on the fitted curves for gosuranemab plasma PK profiles, we then derived the pharmacodynamic effects of CSF free tau reduction^6^.

**Table 1:** Pharmacodynamic effects on CSF protein and accumulated uptake into the neuronal compartment. Where flat doses were used in the clinical trial, they were assumed to be associated with a body weight of 70 kg to arrive at the dose in mg/kg.

Antibody	Kd Mono (nM)	Kd Oligo (nM)	Half-life (h)	CSF mono target engagement	Syn cleft oligomeric target engagement	Max reduction oligomeric neuronal uptake
Tau						
Gosuranemab 30, 60 mg/kg in PSP	0.1	0.1	550–650	99%	2%	< 0.1%
Tilavonemab 30, 60 mg/kg in PSP	20	20	650–880	92%	2%	< 0.1%
Semorinemab 60, 115 mg/kg in AD	3.8	3.8	400	45%	1%	0.3%
a-synuclein						
Cinpanemab 3.5–50 mg/kg	100	0.1	700	6%	95%	97%
Prasineuzumab. 20–60 mg/kg	20	0.05	260	4%	81%	80%

The simulated decrease in free monomeric tau CSF after a single application of different doses of gosuranemab (Fig. 2) fitted well with experimental^6^ data, except for the 3 mg/kg at 85 days post-dose. Using the reported affinity of 100 pM substantially underestimated the response for all doses and time points. Here, the best fit was achieved for a 20 pM affinity of the antibody for monomeric tau. The value of free CSF tau at 84 days for the 3 mg/kg group is grossly underestimated, possibly due to some blood contamination. Given the difference between plasma and CSF levels, a threefold increase (300%) in CSF antibody concentration would need less than 1% contamination from the plasma.

### Effect of antibodies on tau dynamics in different compartments in Phase 2 studies

In contrast to the CSF, which is readily accessible in clinical settings, we wanted to obtain an estimate of the corresponding changes in other inaccessible compartments, i.e., ISF, synaptic cleft compartment and neuronal compartment, important for the proposed mechanism of action. We simulate each of the antibodies with their appropriate dose schedule in their Phase 2 indication.

### Gosuranemab Phase 2 study

**Figure 3:**
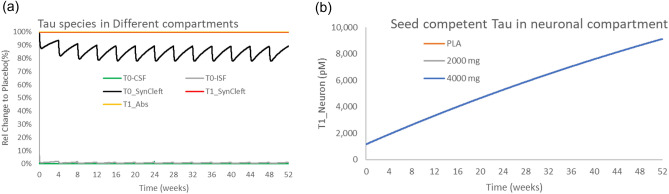
Effect of gosuranemab on tau in different compartments in a 52-week PSP study. Effect of gosuranemab on free tau (**a**) in different compartments at 4000 mg Q4 W. The pharmacology leads to an almost full depletion of free CSF and ISF monomeric and oligomeric tau and a 10–15% decrease in monomeric synaptic cleft tau but no change in synaptic cleft or neuronal uptake of oligomeric tau. (**b**) The reduction in neuronal oligomeric tau uptake by gosuranemab is very small relative to placebo (0.008%).

Gosuranemab was tested in a PSP population for 52 weeks at doses of 2000 mg and 4000 mg^7^. Despite achieving CSF-free tau reductions of over 98%, no clinical improvement was observed. The simulations (Fig. 3) reproduced the observed clinical effects of gosuranemab (i.e., clear target engagement with regard to free CSF tau, but no impact on disease progression). When calculating the area under the curve (AUC) over the whole treatment period for uptake of oligomeric tau in neurons (which takes into account drug exposure and PK profile), there is a small effect (15–20%) on monomeric synaptic cleft tau because of the relatively weak affinity for postsynaptic receptors. On the other hand, due to the avid binding of oligomeric tau to postsynaptic receptors, gosuranemab maximally reduces the uptake of oligomeric tau by 0.008%. Simulating a hypothetical dose of 8100 mg gosuranemab results in a 0.013% decrease.

We also simulated the effect of gosuranemab in a 52-week study in AD patients (Fig. S9), essentially leading to a slightly higher pharmacodynamic effect, i.e., a reduction of 0.4% in accumulated oligomeric tau uptake. No clinical data have been reported, but the clinical development project was halted.

### Tilavonemab phase 2 study

**Figure 4:**
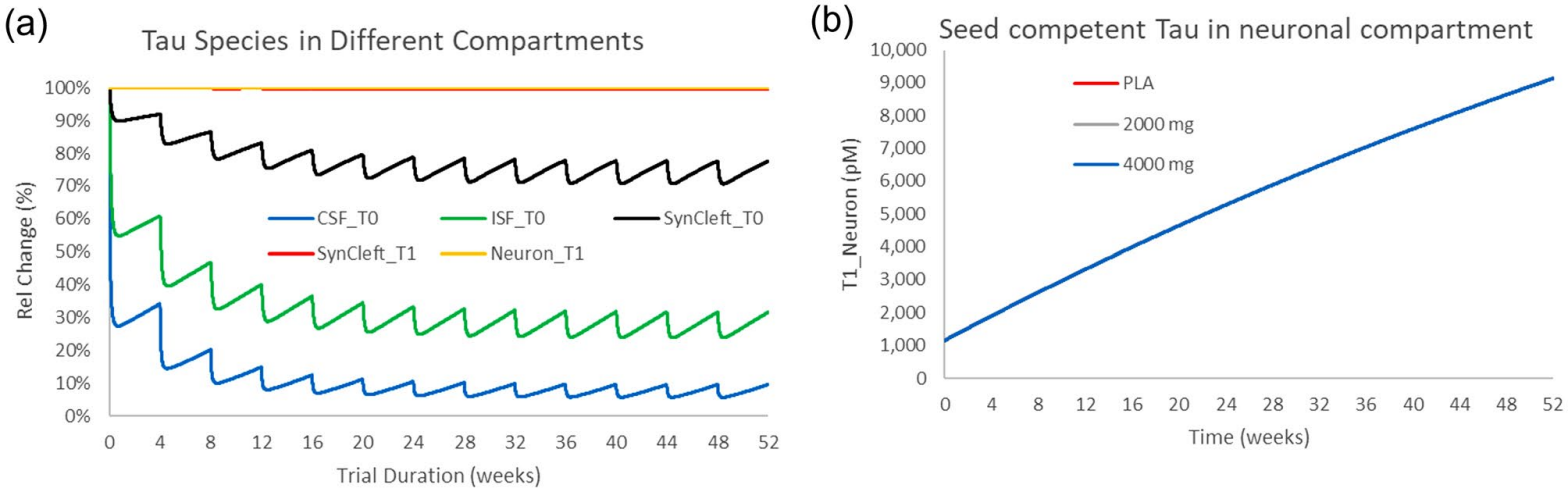
Effect of tilavonemab on free monomeric and oligomeric tau in different compartments in PSP. (**a**) Simulated profiles of free monomeric tau in CSF, ISF and synaptic cleft oligomeric tau for a 52-week tilavonemab study at 4000 mg Q4 W. The level of target engagement decreases substantially from the CSF to the synaptic cleft. (**b**) Dynamics of neuronal oligomeric tau in a 52-week study at 2000 and 4000 mg. Despite good target engagement on free monomeric CSF tau, there was almost no reduction in the uptake of synaptic oligomeric tau (0.01%).

Tilavonemab was tested at doses of 2000 and 4000 mg Q4 W for 52 weeks in a PSP clinical trial^8^. No beneficial treatment effect on clinically relevant scales was found. The simulations (Fig. 4) suggest that neither of the two doses was able to reduce the uptake of synaptic cleft oligomeric tau in these PSP conditions, despite showing reasonable target engagement in the CSF and ISF and on monomeric tau in the synaptic cleft. Even at a hypothetical dose of 8100 mg, neuronal tau uptake will decrease by only 0.36% up from 0.28%.

This suggests that an affinity of 20 nM is likely too low to have a substantial effect on tau uptake. When compared to gosuranemab, tilavonemab can partially compensate for this weak affinity with a much longer half-life.

### Semorinemab Phase 2 study

Semorinemab was tested at doses of 1500, 4500 and 8100 mg in a 73-week study in Alzheimer’s patients without demonstrating significant improvement on the CDR-SOB^9^.

**Figure 5:**
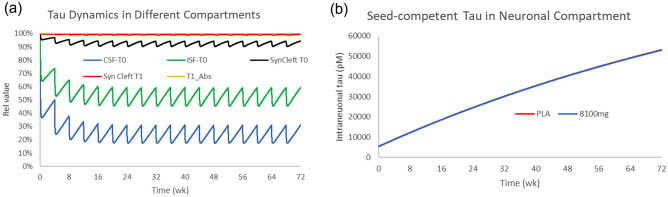
Effect of semorinemab on free monomeric and oligomeric tau in different compartments in AD. Simulated outcomes of free tau dynamics in different compartments in the 8100 mg semorinemab dose Q4 W study for 18 months (**a**) and intraneuronal oligomeric tau dynamics over 73 weeks (**b**). The target engagement of the free monomeric tau is substantial in CSF and ISF and less so in the synaptic cleft; however, the drug can only reduce neuronal uptake of oligomeric tau by 0.3%.

We investigated the effect of these interventions in different compartments as a single endpoint at 73 weeks or as an accumulated change over the whole trial duration (Fig. 5). The simulations show that the drug, despite showing some target engagement in CSF and ISF, is unable to significantly reduce synaptic cleft oligomeric tau (0.38%), possibly explaining the lack of clinical efficacy. This is likely due to the relatively weak affinity (3.6 nM).

The reduction in uptake is slightly higher for semorinemab than gosuranemab in PSP due to the lower affinity of oligomeric tau for postsynaptic receptors in the AD case.

### Effect of aSyn antibodies on aSyn dynamics in different compartments in Phase 2 studies

In this section, we simulate each of the antibodies with their appropriate dose schedule in their Phase 2 indication of Parkinson’s disease.

### Cinpanemab Phase 2 study

**Figure 6:**
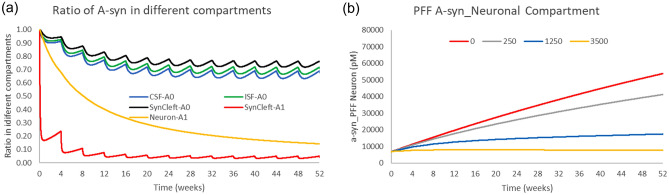
Effect of cinpanemab on free monomeric and oligomeric aSyn in different compartments. Dynamics of synaptic free monomeric and PFF aSyn in different compartments during treatment for 52 weeks (Q4 W) in PD with the highest dose of 3500 mg cinpanemab (**a**). Due to the high selectivity of the antibody for PFF aSyn, the reduction is much more pronounced than for monomeric aSyn. (**b**) Dynamics of neuronal PFF a-Syn uptake with and without cinpanemab at different doses (in mg/kg) during the 52-week study, showing a substantial dose-dependent reduction in neuronal uptake.

The anti-aSyn antibody cinpanemab Q4 W was tested in a 52-week study of Parkinson’s patients at doses of 3.5, 15 and 50 mg/kg corresponding to 250, 1250 and 3500 mg^10^. Simulation of the dynamics of synaptic oligomeric aSyn suggests a substantial reduction in free synaptic PFF aSyn levels. (Fig. 6). The results suggest that cinpanemab can strongly reduce the accumulated neuronal uptake of oligomeric aSyn by 26%, 76% and 97% at doses of 250, 1250 and 3500 mg. The reduction would even be 99% at a hypothetical dose of 8100 mg Q4 W.

### Prasineuzumab Phase 2 study

**Figure 7:**
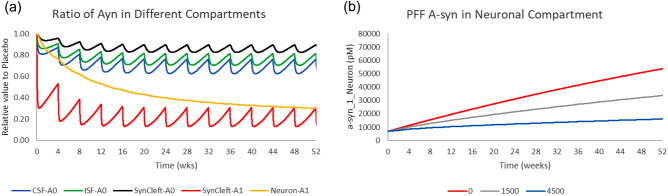
Effect of prazineuzumab on free monomeric and oligomeric aSyn in different compartments. (**a**) Dynamics of monomeric and oligomeric aSyn in different compartments during a 52-week study with 4500 mg Q4 W prazineuzumab in PD. (**b**) Neuronal uptake dynamics for a 52-week study with and without different doses (1500 and 4500 mg) Q4 W of prazineuzumab, showing considerable reduction of PFF aSyn uptake.

Prasineuzumab was tested at doses of 20 and 60 mg/kg corresponding to 1500 and 4500 mg for a trial duration of 52 weeks^11^. This compound has approximately 100-fold oligomeric selectivity over monomeric aSyn. The simulations suggest that free PFF aSyn levels in the synaptic cleft and the uptake of oligomeric aSyn in the afferent neuron by prasineuzumab are substantially reduced (Fig. 7). The model outcomes suggest that the area under the curve for oligomeric aSyn uptake would be reduced by 80% at the highest dose of 4500 mg.

### Sensitivity analysis

We studied the impact of several parameters on the predicted neuronal uptake of oligomeric tau or PFF aSyn (Supplemenetary information).

A key parameter is the flow rate between ISF and the synaptic cleft, which determines the accessibility of antibodies to the compartment of action, the synaptic cleft. In the Supplementary Information, we provide more evidence for the selection of this value, which is driven by the tortuosity and the possible binding to FcgR, internalization and degradation of IgG antibodies in microglia cells.

Increasing the flow rate between ISF and synaptic cleft from the selected value of 0.001 l/h to 0.01 l/h led to a slight decrease in the uptake of neuronal oligomeric protein (Table S3). For tau antibodies, uptake would decrease by maximally 0.3% in PSP and 4% in AD. In contrast, for aSyn antibodies, increasing the flow rate (and availability of the antibodies) would almost completely reduce the uptake of PFF aSyn (> 99%).

Another important parameter is the affinity of oligomeric tau for postsynaptic receptors as they enter in direct competition with the antibodies in the synaptic cleft. Increasing the affinity of PFF aSyn for postsynaptic receptors from the experimental value of 800 nM significantly affected neuronal uptake (Fig. S10). An increase in the affinity of oligomeric aSyn to its postsynaptic receptors (Kd below 50 nM) reduced the effect of Cinpanemab mg from 98 to 73%. Similarly, decreasing the affinity of the antibody for oligomeric protein down to 2 nM (100-fold) decreased the effect on reduction from 98 to 45%. These scenarios might occur when the oligomeric aSyn is sufficiently different from the PFF aSyn.

In contrast, reducing the affinity of oligomeric tau to its receptor in the PSP case from 0.3 nM to 1600 nM improves the effect of 4500 mg gosuranemab, with a reduction in neuronal uptake increasing from < 1% to 35% over that range (see Fig. S11). Additionally, because anti-tau antibodies affect monomeric tau as well, sensitivity analysis of the affinity of monomeric tau for postsynaptic receptors over a range of 20–1600 nM showed that the reduction in oligomeric tau neuronal uptake remained well below 1%.

In summary, the simulations (Table 1) suggest that tau antibodies, despite reaching significant CSF target engagement on free monomeric tau, fail to affect the dynamics of oligomeric tau in the synaptic cleft. As a consequence, they are unable to reduce the uptake of oligomeric tau in the afferent neuron, possibly explaining the lack of effect on clinical progression. Key properties driving this outcome are the lack of selectivity for oligomeric tau, the relatively weak affinity and the dose, in addition to the affinity of oligomeric tau for postsynaptic receptors.

In contrast, the simulations on the seed competent-selective anti-aSyn antibodies suggest that while free monomeric aSyn in the CSF is not affected, the antibodies are able to substantially reduce uptake of PFF aSyn in the afferent neuron. The lack of clinical effect therefore cannot be explained by the current assumptions of the model; in addition to the higher affinity of oligomeric aSyn for postsynaptic receptors (see above), other suggestions will be discussed below.

## Discussion

Tau and aSyn immunotherapies have been of high interest in the AD and PD fields, respectively, based on the hypothesis that these proteins can move from one cell to another in a prion-like fashion^39, 47^. However, recently published clinical data for gosuranemab, tilavonemab, semorinemab, cinpanemab and prasineuzumab have been disappointing.

Epitope location has been hypothesized to be one of the reasons for the failure of some trials^12, 13^. Other explanations include short trial duration, endpoints not appropriate for patients in the early stage of the disease or exclusion of patients starting symptomatic treatments^14^.

We wanted to explore more quantitatively other possible hypotheses to explain the failure of these trials focusing on target engagement and biological mechanisms of protein uptake in the compartment important for the mechanism of action of the drugs.

We therefore have developed a quantitative systems pharmacology model simulating the dynamics of misfolded protein release and uptake by neuronal cells and their interaction with antibodies in different brain compartments. The model used appropriate values for the volumes of key brain compartments, was calibrated using preclinical data for tau and aSyn uptake and quantitatively reproduced the levels of free CSF and ISF monomeric protein, as measured in clinical observational studies. A minimal PBPK model was used to estimate the absolute antibody dynamics in different central compartments.

The main finding of our study was that while high target engagement for monomeric tau can be achieved in the CSF (experimentally accessible) at the highest doses used in the clinic for gosuranemab (99%), tilavonemab (92%), and only partially with semorinemab (45%), the corresponding target engagement levels for oligomeric tau in the synaptic cleft are much lower (< 1%). The lower target engagement of semorinemab in CSF compared to gosuranemab may be due to its lower affinity and shorter half-life. This lack of target engagement in the synaptic cleft may be one of the reasons for their lack of efficacy.

In contrast, high target engagement of aggregated aSyn in the CSF for cinpanemab (100%) and prasineuzumab (99%) leads to a substantial reduction in free aggregated aSyn in the synaptic cleft (81 and 72%, respectively). When considering the reduction in neuronal uptake of oligomeric protein – which is the ultimate driver of neurodegeneration – the effect of the drugs on the AUC over the whole duration of the trial is less than 1% for gosuranemab, tilavonemab and semorinemab, while it is 72 and 65% for cinpanemab and prazineuzumab, respectively.

QSP modelling also allows us to identify possible improvements.

First, increasing the dose of gosuranemab to 8100 mg (~ 115 mg/kg) enhanced the effect on neuronal uptake, but the reduction in tau did not exceed 1%. For cinpanemab and prasinezumab, neuronal aSyn uptake will be reduced by 99% and 89%, up from 97 and 80%, respectively.

Increasing half-life may be another option to increase target engagement. Prasineuzumab, with a short half-life of 260 h, reduced aggregated aSyn uptake by neuronal cells by maximally 80% in the synaptic cleft, while cinpanemab, with a more than twofold longer half-life (700 h), reduced it by 97%, despite a lower dose and affinity.

Another strategy could be to increase antibody brain penetration using receptor-mediated transcytosis. This was done with Roche’s RO7126209 (NCT04639050; NCT04023994), an engineered version of gantenerumab with an Fc-bound Fab fragment binding the transferrin receptor. However, this approach negatively impacts antibody plasma half-life, reported to be three to six days with RO7126209 (https://www.alzforum.org/therapeutics/ro7126209), which can lead to reduced accumulated target exposure.

Second, tilavonemab and semorimenab have relatively modest affinities for both monomeric and oligomeric proteins (20 and 3.6 nM, respectively), resulting in lower target engagement in the synaptic cleft compared to gosuranemab with a Kd of 0.02 nM. Both prasinezumab and cinpanemab with relatively high affinities for PFF α-synuclein (50 and 100 pM) result in synaptic cleft target engagement of 80 and 97% at their highest dose.

Third, the model clearly suggests a role for specificity of the antibody on oligomeric over monomeric protein. Both prazineuzumab and cinpanemab, with high selectivity for aggregated aSyn (400- to 1000-fold), result in substantial synaptic cleft target engagement. This is essential to compensate for the limited brain penetration of antibodies to fully engage with pathological proteins. Such high selectivity can reduce “off-target” binding to nonseeding species. To date, none of the clinically tested anti-tau antibodies have demonstrated such specificity, with the notable exception of zagotenumab (MC1), which claimed to bind a conformation-specific epitope, but this antibody has a modest affinity (5–10 nM). Specificity for aggregated species, at least the PFF form, can be achieved quite readily for aSyn, as demonstrated by cinpanemab^48^ and prasineuzumab^31^ and for a number of anti-beta-amyloid antibodies. Despite recent cryo-EM descriptions of pathological tau and aSyn from AD and PD postmortem brains^49^, the exact identities of seeding species are not yet all identified, making this approach difficult.

Another important point for immunotherapy efficacy is the accessibility of the antibody to the synaptic cleft. The simulations suggest synaptic cleft antibody levels to be in the 20–50 pM range, which would correspond to 0.5–2.5 molecules/synapse. In contrast, the number of free tau or aSyn molecules ranges between 5 and 20 molecules/synapse. Together with the high density of postsynaptic “acceptors”, there is ample opportunity to escape capture by antibodies. Although not quantitatively documented, similar concerns have recently been raised in the literature^50^.

Accessibility of antibodies to the synaptic cleft depends to a great extent on the diffusion of the antibody in the ISF (quantified by tortuosity) and the dimensions of the synaptic cleft (for a review see^51^). In addition, as shown in the Supplementary Information, futile binding of the antibody to FcgR on microglia can substantially decrease concentration levels in the ISF. In that regard, it is worth noting that semorinemab with effector function^52^ had the same effect on tau propagation in preclinical studies as the effector-less version, suggesting a limited role for microglia in removing tau-antibody complexes. Therefore, having an antibody with minimal binding to FcgR on microglia should be preferred.

Furthermore, with the longest dimension of an IgG antibody being approximately 13–14 nm^53^, not much smaller than the synaptic cleft width (20–40 nm)^54^ where transsynaptic cell adhesion molecules such as neurexins, neuroligins, laminins, lecticans, tenascin-C and hyaluronic acid form a dense extracellular matrix^55^, it is conceivable that the probability of an antibody encountering a tau or an aSyn molecule is even more limited^56, 57^. In addition, there is growing evidence that the release of tau, hijacking presynaptic release vesicles^58^, is confined to nanocolumns that align presynaptic hotspots with a higher density of postsynaptic receptors^59^. The reduced accessibility of therapeutic interventions might be partially mitigated by using single chain Fv antibodies or nanobodies, but they usually have shorter half-lives^50^.

Notably, the case of beta-amyloid antibodies in AD is somewhat different, as their target is a large extracellular beta-amyloid plaque, typically 20–40 µm in diameter^60^ with multiple epitopes (with the exception of solaneuzumab) , and the engagement of the antibodies with microglia drives the amplification of the biomarker response. In addition, the presence of these plaques likely disrupts the brain parenchyma, leading to a 10–15% increase in extracellular fluid, at least in APP23 beta-amyloid Tg mice^61^.

Somewhat related to this issue is the shuttling of oligomeric tau in extracellular vesicles^62^; in principle, we can assume that this fraction is inaccessible to the antibodies. Different tauopathies seem to use this process to different degrees^62^, allowing us to simulate the impact of antibodies in different diseases.

The competition of antibodies with the postsynaptic binding sites HSPG or LRP1 for the oligomeric protein, based on their affinity and density^40^, also determines the pharmacodynamic effect on neuronal uptake. Affinities of oligomeric protein for HSPG or LRP1^21^ are much lower than antibodies, but tau extracted from PSP patients brain extracts have been documented to internalize more rapidly compared to AD patients^23^, making it more challenging to develop these antibodies for PSP.

There are a number of differences in the biology of tau and aSyn uptake. Oligomeric tau interaction with HSPG heavily depends upon specific glycosaminoglycan lengths and sulphate moiety positions, while aSyn aggregates are less sensitive to these modifications^63^. In addition, based on the difference in CSF levels, the model predicts that secretion and uptake dynamics tend to be different. A lower uptake is a consequence of the much lower affinity of PFF for the postsynaptic receptor (800 nM) compared to oligomeric tau from AD (40 nM) and PSP (0.3 nM).

Unlike the situation with the current anti-tau antibodies, aSyn antibodies are predicted to reduce PFF aSyn neuronal uptake in the 80–100% range, suggesting that the reason for clinical trial failure might be due to other factors beyond accessibility. ASyn remains a rational target for Parkinson’s disease^64^, as genomic duplication or triplication of the aSyn gene causes hereditary early-onset parkinsonism with dementia^65^

While it is conceivable that uptake of neuronal oligomeric aSyn is not associated with clinically detectable neuronal dysfunction, a more likely explanation is that the oligomeric aSyn in Parkinson’s disease differs substantially from the preformed fibrils used in research and therefore interacts less with the antibodies. Additional concerns relate to the effect size and the time scale between uptake of oligomeric aSyn and its impact on functional neuronal circuit behaviour that translate to clinical scales. aSyn antibodies may also need to reduce monomeric aSyn uptake. Endpoints used may not be sensitive enough at this earlier disease stage.

There are a number of limitations to the model. First, we assume that antibodies do not enter the neuronal compartment to engage the relevant intracellular protein, despite reports that certain forms are taken up in the neuronal compartment^66^. Second, we did not include any detailed effect of microglia-dependent tau or aSyn clearance. There is evidence that microglia have a complex role in tau clearance, ranging from degradation to enhancement of spreading^67^; on the other hand, microglia are capable of phagocytosing aSyn^68^ or can spread the pathology^69^. Third, the model assumes only neuron-to-neuron transmission of misfolded proteins via the synaptic cleft. While this likely refers to the majority of the transmission, there is some evidence that misfolded protein can diffuse out the synaptic cleft and enter the neuron along the axonal projections, at least in neuronal cell culture experiments with microfluidic chambers^70^. This process might be enhanced by the demyelinization often observed in neurodegenerative disorders. Fourth, the model readout is limited to the uptake dynamics of monomeric and oligomeric protein in the neuronal compartment and does not explicitly take into account subsequent steps, i.e. the oligomerization kinetics of the proteins. While this is an important driver of the pathology, we assume that the amount of aggregated forms that over time can be detected as NFT or Lewy bodies is proportional to the concentration of the oligomers that enter the neuronal compartment. Fifth, the secretion rate of proteins is fixed and adjusted so that an average CSF value is consistent with clinical observations. However, secretion is an active process and proportional to neuronal activity^71^, which itself might be related to beta-amyloid-induced hyperactivation, certainly in early AD^72^. Later versions of the model can take this relationship into account or include specific intracellular oligomerisation, opening the possibility to develop a more extensive beta-amyloid-tau QSP model.

To our knowledge, our study is the only one comparing five tau and aSyn antibodies with published clinical data and may help future selection of tau and aSyn therapeutic modalities and clinical trial dose selection. We are aware of only one other report modelling the impact of anti-tau antibodies on tau dynamics in humans^73^; however, this model does not include the biological processes driving tau dynamics in the synaptic cleft and the neuronal compartment.

## Conclusions

In summary, this model aims to develop a quantitative and mechanistic understanding of the target engagement of tau and aSyn antibodies and their impact on the progression of AD and PD pathology based on capturing the oligomeric protein in the synaptic cleft. Using biologically relevant parameters, we generated hypotheses for the lack of efficacy of current clinical trials and highlighted specific properties of antibodies that could lead to more successful interventions.

### Supplementary Information

Supplementary Information 1.

Supplementary Information 2.

## Data Availability

All data and equations of the model are available in the main text or the supplementary materials.

## Abbreviations

AD: Alzheimer’s disease
Asyn: Alpha-synuclein
AUC: Area-under-the-curve
BBB: Blood–brain barrier
CSF: Cerebro -spinal fluid
ISF: Interstitial fluid
HSPG: Heparan sulfate proteoglycans
LRP1: Low density lipoprotein receptor related protein-1
PBPK: Physiology-based pharmacokinetic modeling
PD: Parkinson’s disease
PFF: Preformed (alpha-synuclein) fibrils
PSP: Progressive supranuclear palsy
QSP: Quantitative systems pharmacology
