# Supplementary material for: Analysis of clinical failure of anti-tau and anti-synuclein antibodies in neurodegeneration using a quantitative systems pharmacology model
Source: Sci Rep. 2023 Sep 1;13:14342. doi: 10.1038/s41598-023-41382-0 (PMC10474108; doi:10.1038/s41598-023-41382-0)
Supplement: Supplementary file 2 — Supplementary Information 2. [file 41598_2023_41382_MOESM2_ESM.pdf]

Supplementary information to

Analysis of clinical failure of anti-tau and anti-synuclein antibodies in neurodegeneration using a quantitative systems pharmacology model

Hugo Geerts[1]*, Silke Bergeler[1,4], Mike Walker[2], Piet H. van der Graaf[2], Jean-Philippe Courade[3]*

Calibration of the QSP model for tau

This section addresses the calibration of the uptake parameters of tau into the afferent neuronal compartment. This calibration is based on in vitro cell culture experiments, and we assume that monomeric tau is taken up by the same processes as seed-competent or oligomeric tau.

Fig. S1 shows the similarity between experimentally determined uptake of fluorescent oligomeric tau in human IPSC neurons as a function of time and for different starting concentrations [1].

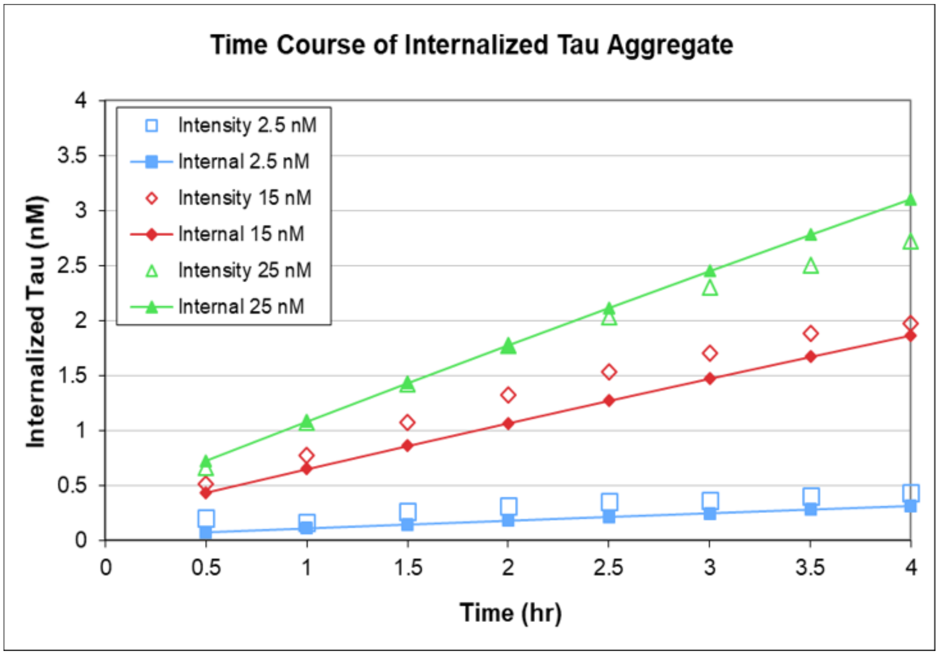

Fig. S1. Calibration of the QSP model to experimental in vitro data. Uptake of fluorescently labelled tau oligomers in hIPSC cells is modelled to derive uptake parameters. Open symbols are experimentally determined values from fluorescent intensity, and closed symbols are predicted model outcomes.

The study in Fig. S2 used primary rat neuronal cultures to which tau-enriched brain extracts from patients with Alzheimer's disease (AD), progressive nuclear palsy (PSP) and cortico-basal degeneration (CBD) were added [2]. Aggregated tau complexes are detected using immunofluorescence.

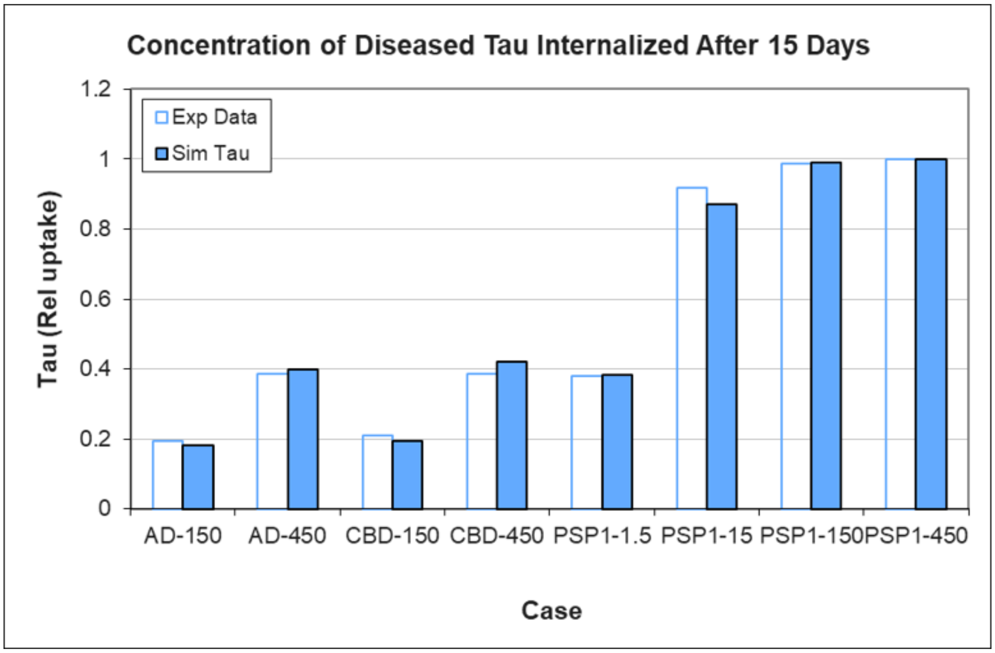

Fig. S2. Calibration of tau QSP model uptake parameters to experimental data from internalization of brain extracts. The model is applied to tau-enriched brain extracts from AD and PSP patients in rat primary neuronal cultures to derive appropriate uptake parameters. AD-150: 150 ng tau; AD-450: 450 ng tau, CBD-150: 150 ng tau, PSP 1-1.50 ng tau, PSP 1-15: 15 ng tau, PSP 1-150: 150 ng tau, PSP 1-450: 450 ng tau [2]

Table S1. Parameters of the model fitting. Experimental observations of in vitro datasets for uptake of different forms of seed-competent tau (brain extracts from AD, PSP and CBD) [2] and fluorescent tau oligomers [1].

**Parameter**	AD	CBD	PSP
Receptor Concentration (nM)	10000	10000	10000
Recovery Rate of Receptors (per min)	1	1	1
Affinity of monomeric tau with receptor (nM)	800	800	800
Affinity of seed-competent tau with receptor (nM)	40	40	0.3
Absorption Rate (per min)	0.01	0.01	0.01

Calibration of the QSP model for a-synuclein

Fig. S3 shows the fitting of the model for uptake of fluorescently labelled preformed fibrils (PPF) of a-synuclein in primary neuronal cultures [3].

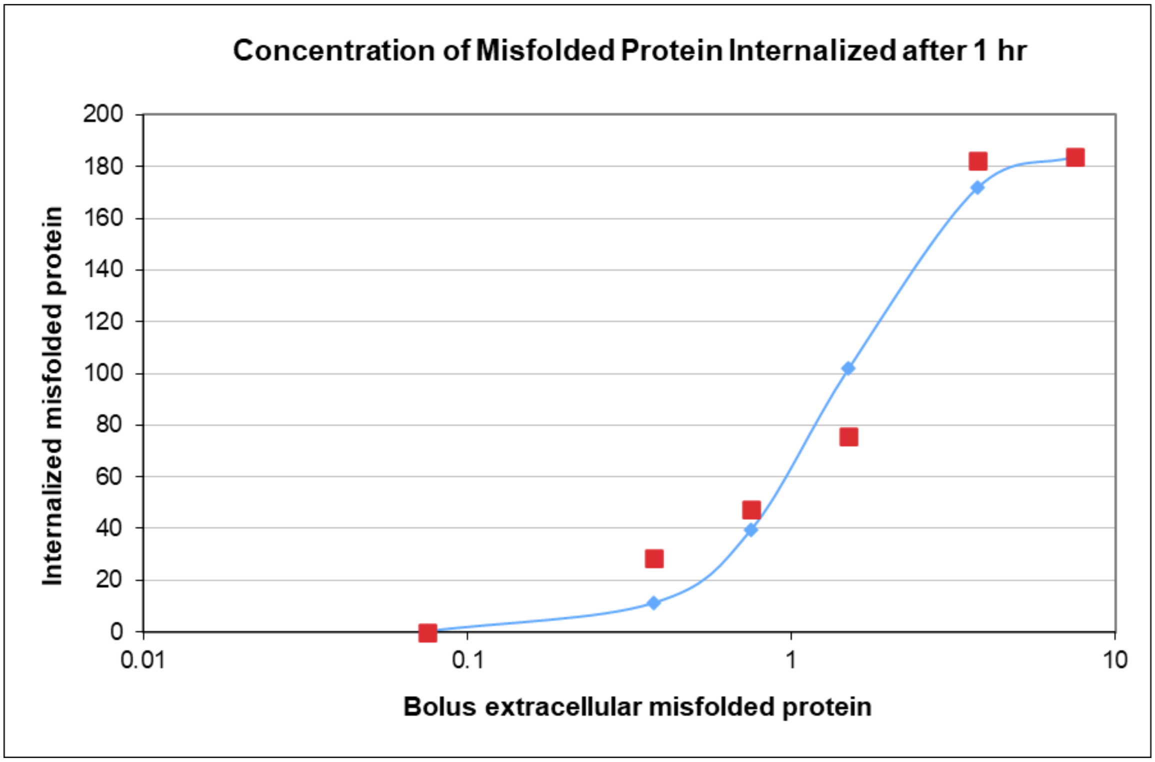

Fig. S3. Calibration of the QSP model for a-synuclein oligomers. Uptake of fluorescently labelled a-synuclein oligomers in hIPSC cells is modelled to derive appropriate values. The X-axis is in nM, and the Y-axis is in A.U.

The best fit was obtained for an absorption rate of 0.5 nM/hr, a receptor density of 10,000 nM and an affinity of PFF a-synuclein of 800 nM for the postsynaptic receptors.

PBPK models for antibodies

In this section, we describe the plasma PK profiles of the different antibodies with the PBPK model, resulting in the following calibration

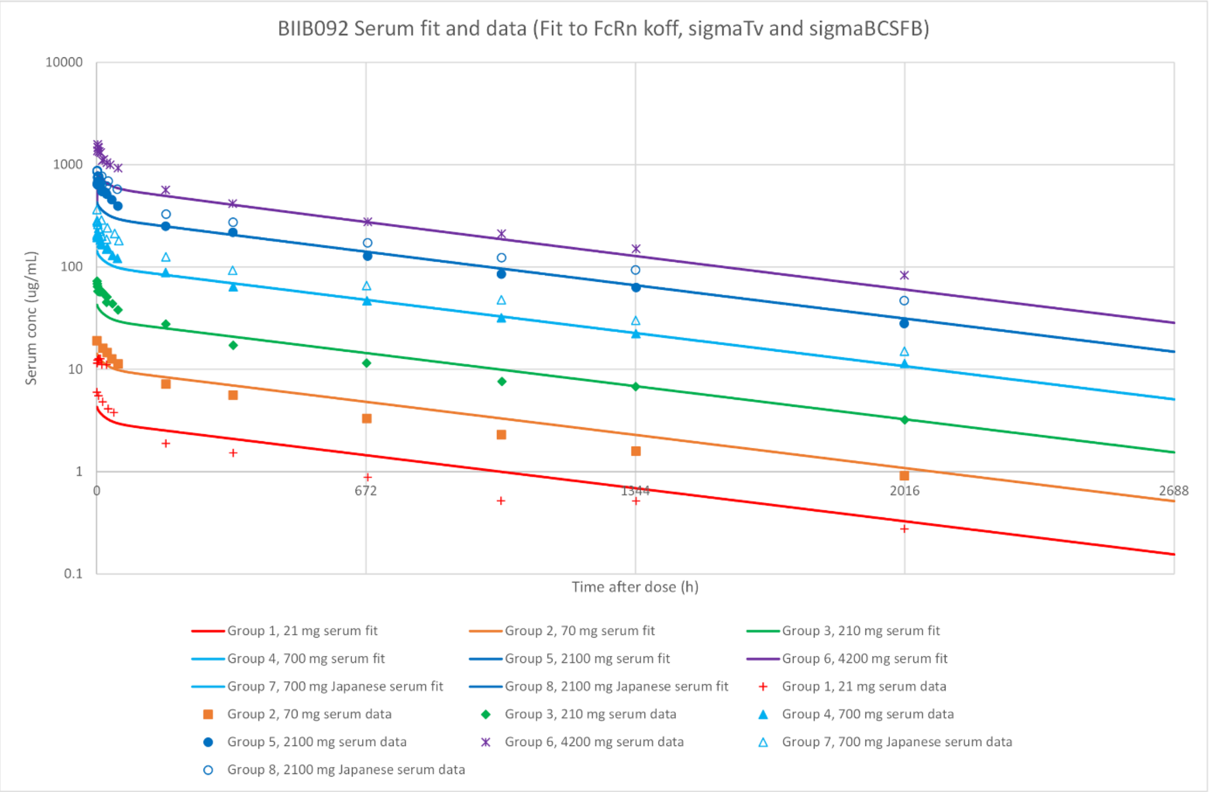

Fig. S4. PK profile of Gosuranemab. Different doses of gosuranemab from the observed Phase 1 study [4] are fitted to derive appropriate PK parameters. Symbols are experimentally determined plasma levels; full lines are predicted values. The model fit does not predict the first days very well, but the fit is significantly better for the later time points, as they drive the accumulated pharmacodynamic effect on tau dynamics.

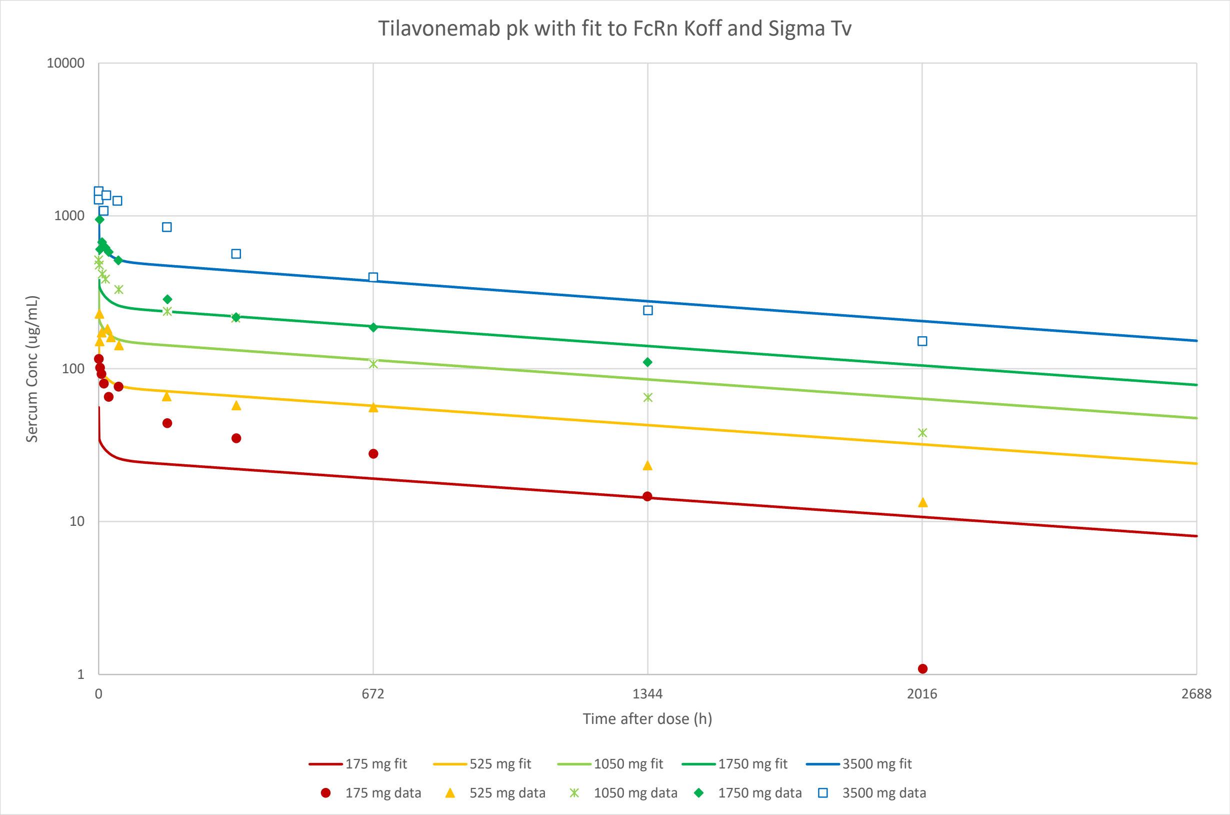

Fig. S5. PK profile of Tilavonemab. Different doses of tilavonemab from the observed Phase 1 study [5] are fitted to derive appropriate PK parameters. Symbols are experimentally determined plasma levels; full lines are predicted values. The model fit does not predict the first days very well, but the fit is significantly better for the later time points, as they drive the accumulated pharmacodynamic effect on tau dynamics.

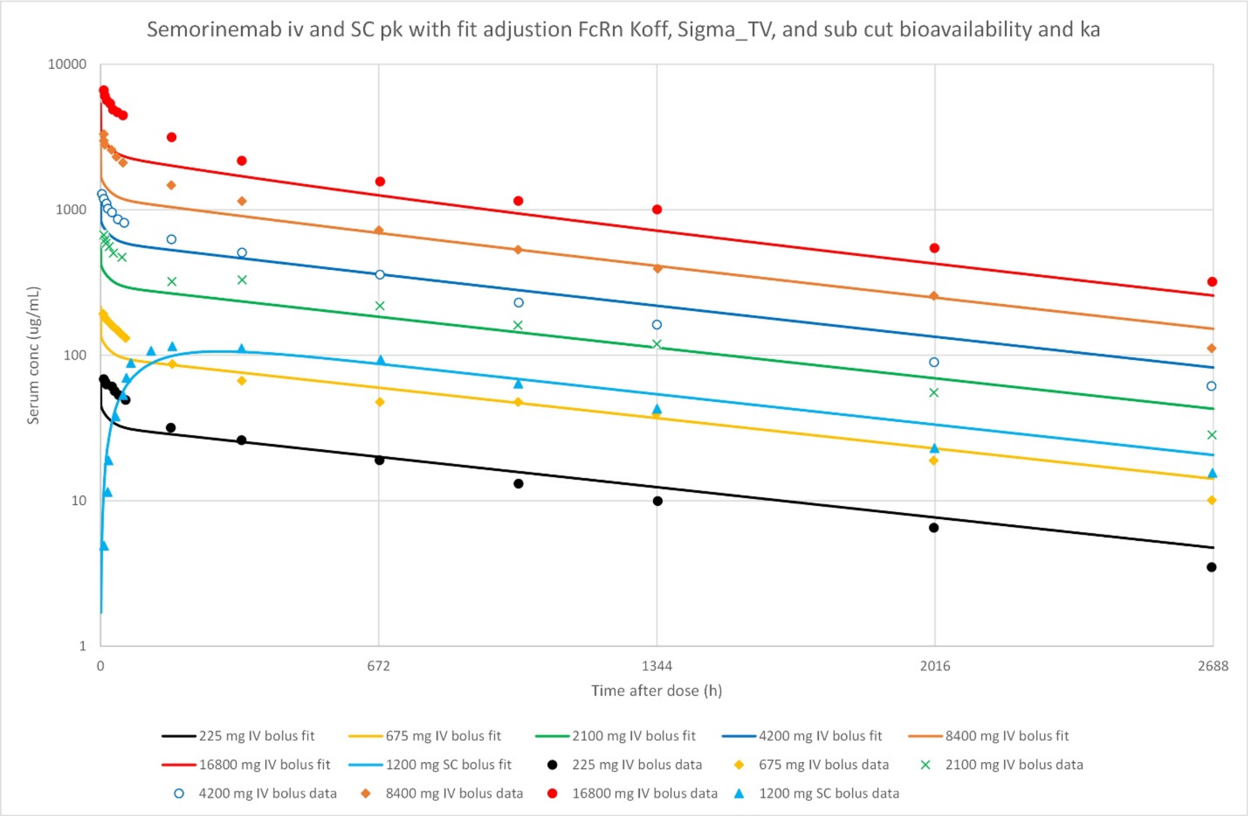

Fig. S6. PK profile of Semorinemab. Different doses of Semorinemab from the observed Phase 1 study [6] are fitted to derive appropriate PK parameters. Symbols are experimentally determined plasma levels; full lines are predicted values. The model fit does not predict the first days very well, but the fit is significantly better for the later time points, as they drive the accumulated pharmacodynamic effect on tau dynamics. Note that we additionally fitted a subcutaneous formulation of the same drug.

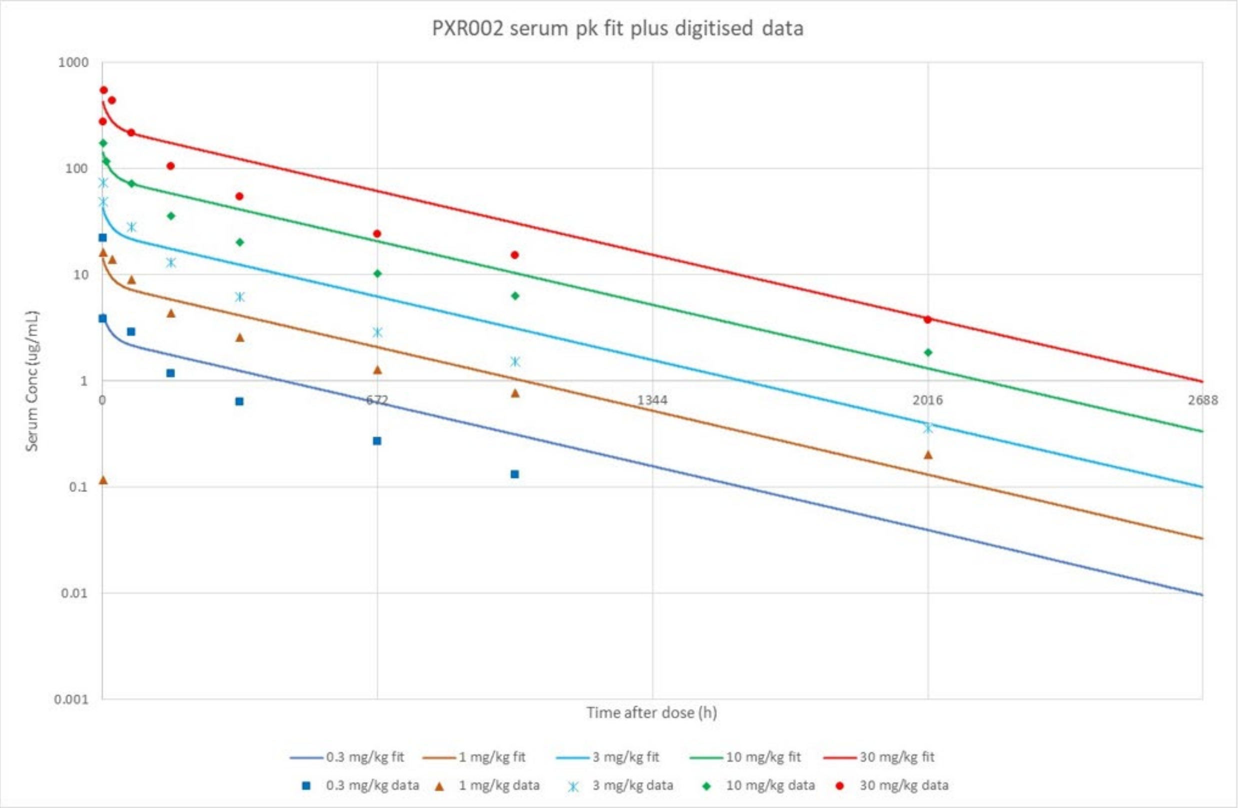

Fig. S7. PK profile of Prazineuzumab. Different doses of prazineuzumab from the observed Phase 1 study [7] are fitted to derive appropriate PK parameters. Symbols are experimentally determined plasma levels; full lines are predicted values. The model fit does not predict the first days very well, but the fit is significantly better for the later time points, as they drive the accumulated pharmacodynamic effect on tau dynamics.

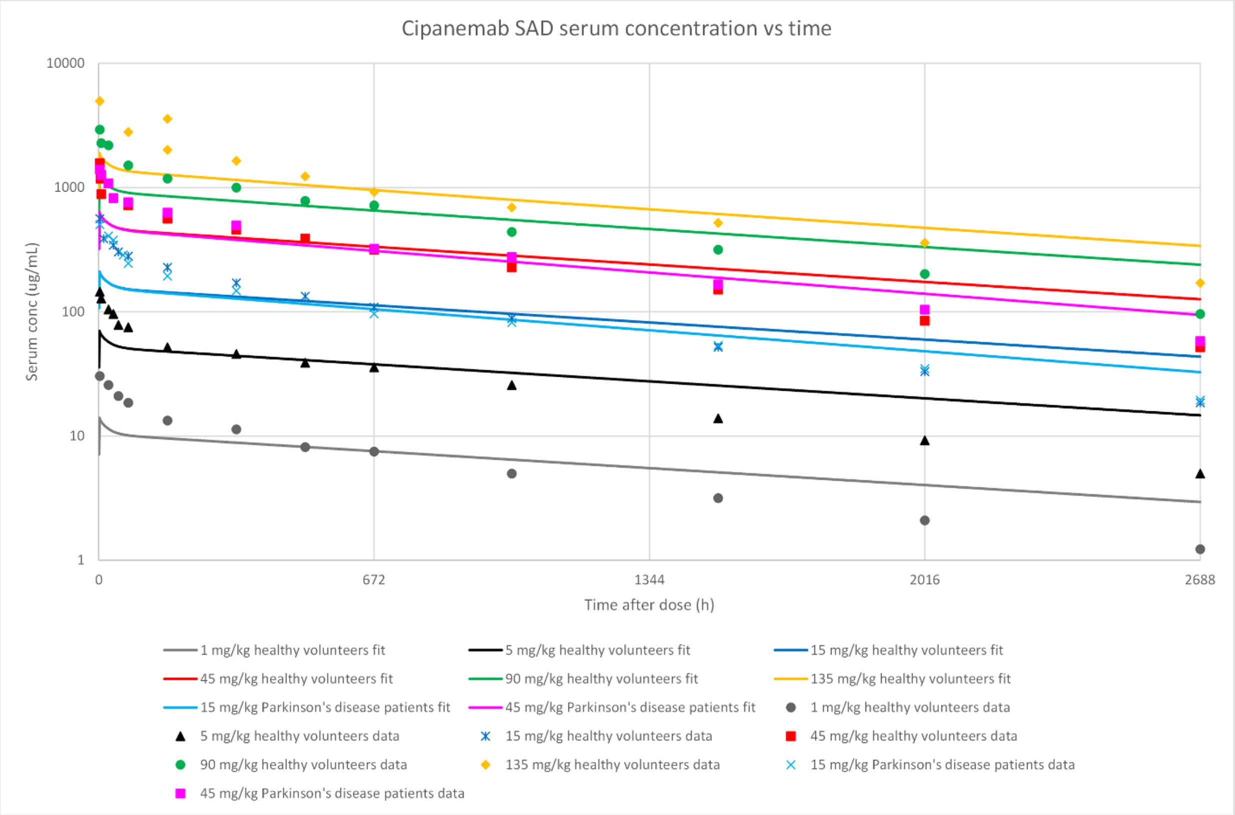

Fig. S8. PK profile of Cinpanemab. Different doses of Cinpanemab from the observed Phase 1 study [8] are fitted to derive appropriate PK parameters PK profiles. Symbols are experimentally determined plasma levels; full lines are predicted values. The model fit does not predict the first days very well, but the fit is significantly better for the later time points, as they drive the accumulated pharmacodynamic effect on tau dynamics.

Table S2. List of parameters after fitting the PK profiles. Koff-FcRn is the off-rate of the antibody from the FcR; sigma BCSFB is the dimensionless reflection coefficient at the Brain-CSF barrier, sigma_T_v is the tissue vascular reflection coefficient.

Antibody	koff_FcRn (1/hr)	sigma_BCSFB (dimensionless)	sigma_T_v (dimensionless)	Reference to PK profile
Gosanerumab	20.7	0.99749	0.9845	[4]

Tilavonemab	7.47	0.9973	0.99	[5]
Semorinemab	12.98	0.9973	0.98	[6]
Cinpanemab	8.13	0.999	0.999	[8]
Prazineuzumab	21.3	0.999	0.843	[9]

Accessibility of the antibodies to the synaptic cleft

A major challenge for any anti-tau or anti-aSyn antibody to impact the pathological trajectory is the accessibility of the antibody to the synaptic cleft, a very restricted space. The antibody first diffuses through the brain parenchyma, a dense collection of various cell types where the extracellular space (usually between 15 and 20% of the brain volume) consists of aqueous channels with a diameter of 65 nm [10]. The diffusion of molecules can be described by the tortuosity l.

Diffusion in the parenchyma is inversely proportional to the square of the tortuosity, which has been measured as 6.0 for an IgG antibody, at least in tumour tissue [11], reducing diffusion by a factor of 36. The effective diffusion can be further reduced substantially by binding of the antibody to nontarget sites, such as FcgR on activated microglial cells. This is especially important for IgG1 antibodies. For example, effective diffusion for lactoferrin and transferrin (both 80 kDa) differs by an order of magnitude as lactoferrin binds extensively to HSPG [12].

We first estimated the diffusion coefficient for diffusion in and out of a synaptic cleft with typical dimensions of 100,000 nm^2 surface and 20-40 nm wide [13] using the equation

$$D_{eff}^{*} = \frac{D_{eff}}{1 + \frac{[T] * Kd}{(Kd + [Ab])^2}}$$

where [T] and [Ab] are the tau and antibody concentrations, respectively, and Kd is the affinity of the antibody for tau. Together with the impact of tortuosity, the results suggest that D*eff is between 0.1 and 0.8% of Deff. This has a substantial impact on the diffusibility of the therapeutic antibody.

Using the Equation $x^2 = 6Dt$, with Deff = $58*10^{-8}$ cm^2/sec [14] for an IgG antibody in aqueous solution, the spatial extension after a single pulse at t = 0 will be 10-11-fold lower when considering the reduced diffusion coefficients. After 1 month, the spatial distance covered by the pulse was reduced from 19 mm to 1.8 mm.

In addition, IgG1 antibodies can bind to FcyR on microglia and be eliminated before they reach the synaptic cleft. The concentration profiles follow the Equation C/C0 = Exp(-x*SQRT(K2/D)) [14]. When applying this formula, it turns out that over the first 60 µm (the average distance between the vascular compartment and neurons) doubling the clearance rate of antibody results in a five-fold lower concentration.

Gosuranemab in AD study

Gosuranemab was tested in an AD population for 52 weeks at a dose of 2100 mg [15]. The drug achieved substantial CSF-free Tau decreases (0 to 98%). The trial was halted due to futility.

A **B**

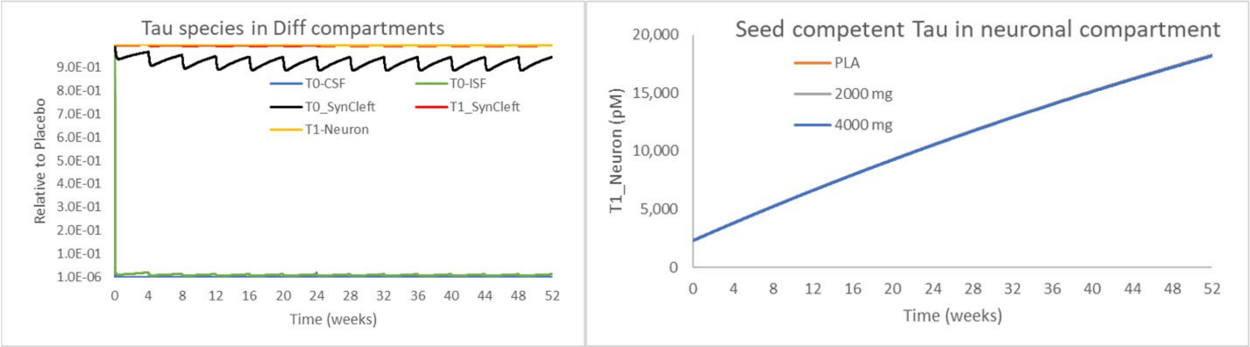

Fig. S9. Gosanerumab in Alzheimer's disease. (a) Effect of gosuranemab on free CSF Tau. (a) in different compartments and synaptic cleft seed-competent tau (B) for a 52-week study at 60 mpk Q4 W in AD patients. The original pharmacology where the affinity for monomeric and seed-competent tau is identical (20 pM) leads to an almost full depletion of free CSF and ISF monomeric and seed-competent tau but almost no change in synaptic cleft monomeric and seed-competent tau. (b) The effect of gosuranemab on neuronal seed-competent au uptake is very limited (0.3% reduction at the highest dose).

The simulations indeed show the large difference in target engagement between the different compartments. When calculating the area under the curve (AUC) over the whole treatment period for uptake of seed-competent tau in neurons – which takes into account drug exposure and PK profile), gosuranemab reduces this outcome by only 1.5%. Simulating a hypothetical dose of 8100 mg results in a 24% decrease for gosuranemab, up from 3%.

Sensitivity analysis of the ISF to the synaptic cleft flow rate factor

Here, we explore the effect of the ISF on the synaptic cleft flow rate factor over a range of 0.1 to 1 L/hr on the antibody-mediated reduction in neuronal uptake of seed-competent protein. The simulations suggest that increasing the fraction of antibodies reaching the synaptic cleft tenfold has a smaller impact (between 4- and 6-fold for anti-tau antibodies and between 5 and 10% for anti-Asyn antibodies) on the total reduction in neuronal uptake.

Table S3. Sensitivity analysis of changes in ISF to SynCleft flow. Impact of changes on the reduction of seed-competent tau and PFF aSyn uptake, measured as the area under the curve (AUC) and normalized to the placebo case. The simulations suggest that these changes do not substantially affect the pharmacodynamic effect of the antibodies on neuronal uptake.

Trial	Flow 0.001 L/hr	Flow 0.005 L/hr	Flow 0.01 L/hr
Gosuranemab 60mpk PSP	0.008%	0.06%	0.3%
Gosuranemab 60mpk AD	0.4%	1.83%	3.79%
Tilavonemab 60 mpk PSP	0.008%	0.018%	0.021%
Semorinemab 115mpkAD	0.38%	1.18%	1.28%
Cinpanemab 50mpk PD	97.6%	99.2%	99.8%
Prazineuzumab 60mpk PD	80%	97%	99.0%

Somewhat related to this issue is the half-life of tau when considering diffusion out of the synaptic cleft and appearing in the ISF and subsequently in the CSF. We expect this half-life to be very long because of (1) the great concentration difference between intraneuronal tau (micromolar) and CSF tau (picomolar), (2) the observation that tau uses the synaptic vesicle release mechanism [16], (3) the presence of "physical" nanocolumns aligning presynaptic hotspots of release with postsynaptic receptors and (4) based on the diffusion coefficient of 40 μm^2/sec [17], the fact that tau molecules can reach the postsynaptic membrane in approximately 10 usec.

Sensitivity analysis of the affinities of monomeric and seed-competent proteins for the postsynaptic membranes

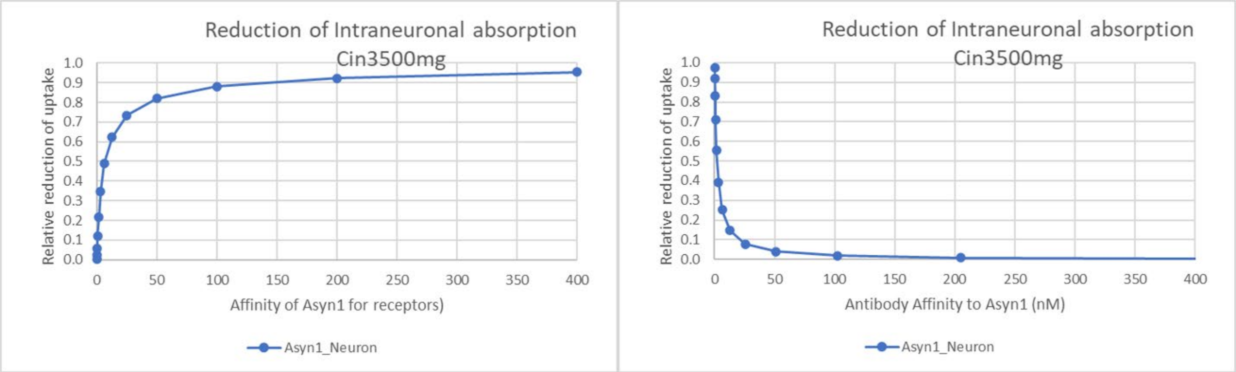

Fig. S10. Changes in affinity of PFF Asyn to postsynaptic receptors. (a) Impact of affinity of PFF aSyn for postsynaptic receptors on PFF aSyn uptake reduction by cinpanemab 3500 mg, measured as the area under the curve (AUC) and normalized to the placebo case. The data show

that increasing the affinity from the experimentally determined value of 800 nM leads to substantially reduced neuronal uptake with a steep drop for affinities higher than 10 nM. (b) Impact of the affinity of cinpanemab for seed-competent aSyn on the reduction in neuronal uptake. Lowering the affinity of the antibody for the seed-competent Asyn reduces the effect with a steep drop from 2 nM upwards.

The simulations suggest that the observed effect of aSyn antibodies on neuronal uptake of PFF Asyn is heavily dependent upon both the affinity of the PFF aSyn for the postsynaptic receptors (threshold affinity higher than 10 nM) and the affinity of the antibody for seed-competent Asyn (threshold affinity lower than 2 nM).

In contrast to the case with aSyn antibodies, decreasing the affinity of oligomeric tau to its receptors from the experimentally observed 40 nM only marginally improves the reduction of neuronal oligomeric tau uptake by Gosuranemab 4500 mg.

Since anti-tau antibodies also strongly bind to epitopes on monomeric tau, we also studied the impact of gosanerumab on neuronal uptake of oligomeric tau as a function of the affinity of monomeric tau for postsynaptic receptors.

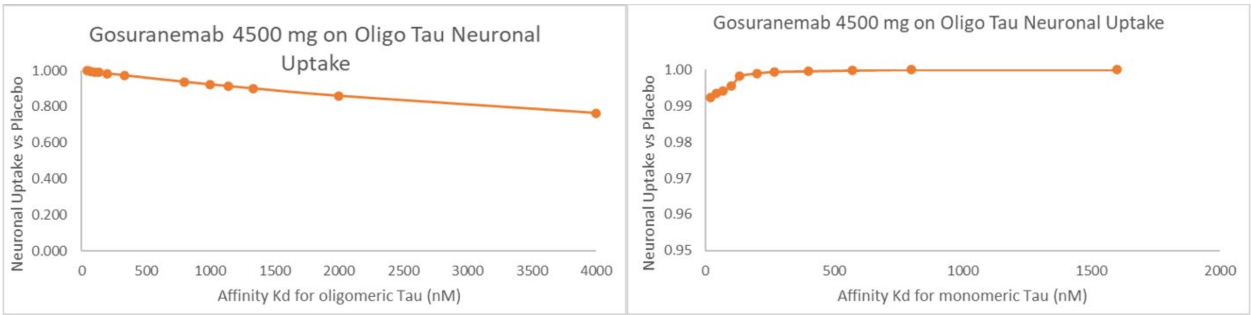

Fig. S11. Change in affinity of oligomeric tau. (a) Impact on reduction in oligo tau neuronal uptake by gosuranemab 4500 mg as a function of the affinity of ***oligo tau*** for postsynaptic receptors, measured as the area under the curve (AUC) and normalized to the placebo case. The data show that decreasing the affinity from the experimentally determined value of 40 nM leads

to a limited reduction in neuronal uptake. (b) Impact on reduction of oligo tau neuronal uptake reduction by gosuranemab 4500 mg as a function of the affinity of ***monomeric tau*** for postsynaptic receptors. The data show that changing the affinity from the experimentally determined value of 800 nM does not affect the subsequent reduction in oligomeric tau uptake.

References

1. Evans LD, Wassmer T, Fraser G, Smith J, Perkinton M, Billinton A, et al. Extracellular monomeric and aggregated tau efficiently enter human neurons through overlapping but distinct pathways. Cell Rep. 2018;22:3612-24.
2. Narasimhan S, Guo JL, Changolkar L, Stieber A, McBride JD, Silva LV, et al. Pathological tau strains from human brains recapitulate the diversity of tauopathies in nontransgenic mouse brain. J Neurosci. 2017;37:11406-23.
3. Karpowicz RJ, Jr., Haney CM, Mihaila TS, Sandler RM, Petersson EJ, Lee VM. Selective imaging of internalized proteopathic alpha-synuclein seeds in primary neurons reveals mechanistic insight into transmission of synucleinopathies. J Biol Chem. 2017;292:13482-97.
4. Qureshi IA, Tirucherai G, Ahlijanian MK, Kolaitis G, Bechtold C, Grundman M. A randomized, single ascending dose study of intravenous BIIB092 in healthy participants. Alzheimers Dement (N Y). 2018;4:746-55.
5. West T, Hu Y, Verghese PB, Bateman RJ, Braunstein JB, Fogelman I, et al. Preclinical and clinical development of ABBV-8E12, a humanized anti-tau antibody, for treatment of Alzheimer's disease and other tauopathies. J Prev Alzheimers Dis. 2017;4:236-41.

6. Ayalon G, Lee SH, Adolfsson O, Foo-Atkins C, Atwal JK, Blendstrup M, et al. Antibody semorinemab reduces tau pathology in a transgenic mouse model and engages tau in patients with Alzheimer's disease. Sci Transl Med. 2021;13:eabb2639.
7. Schenk DB, Koller M, Ness DK, Griffith SG, Grundman M, Zago W, et al. First-in-human assessment of PRX002, an anti-alpha-synuclein monoclonal antibody, in healthy volunteers. Mov Disord. 2017;32:211-8.
8. Brys M, Fanning L, Hung S, Ellenbogen A, Penner N, Yang M, et al. Randomized phase I clinical trial of anti-alpha-synuclein antibody BIIB054. Mov Disord. 2019;34:1154-63.
9. Sopko R, Golonzhka O, Arndt J, Quan C, Czerkowicz J, Cameron A, et al. Characterization of tau binding by gosuranemab. Neurobiol Dis. 2020;146:105120.
10. Sykova E, Nicholson C. Diffusion in brain extracellular space. Physiol Rev. 2008;88:1277-340.
11. El-Kareh AW, Braunstein SL, Secomb TW. Effect of cell arrangement and interstitial volume fraction on the diffusivity of monoclonal antibodies in tissue. Biophys J. 1993;64:1638-46.
12. Thorne RG, Lakkaraju A, Rodriguez-Boulan E, Nicholson C. In vivo diffusion of lactoferrin in brain extracellular space is regulated by interactions with heparan sulfate. Proc Natl Acad Sci U S A. 2008;105:8416-21.
13. Junge W, McLaughlin S. The role of fixed and mobile buffers in the kinetics of proton movement. Biochim Biophys Acta. 1987;890:1-5.
14. Wolak DJ, Thorne RG. Diffusion of macromolecules in the brain: implications for drug delivery. Mol Pharm. 2013;10:1492-504.

15. Dam T, Boxer AL, Golbe LI, Hoglinger GU, Morris HR, Litvan I, et al. Safety and efficacy of anti-tau monoclonal antibody gosuranemab in progressive supranuclear palsy: a phase 2, randomized, placebo-controlled trial. Nat Med. 2021;27:1451-7.
16. Tracy TE, Madero-Perez J, Swaney DL, Chang TS, Moritz M, Konrad C, et al. Tau interactome maps synaptic and mitochondrial processes associated with neurodegeneration. Cell. 2022;185:712-28.e14.
17. Zheng K, Jensen T, Scatchenko L, Levitt J, Suhlinf K, Rusakov D. Nanosclae diffusion in the synaptic cleft and beyond meausred with time-resolved fluorescence anisotropy imaging Sci Reports, 7, 42022, 2017.

Additional files

Fig. S1. Calibration of the QSP model to experimental in vitro data. Uptake of fluorescently labelled tau oligomers in hIPSC cells is modelled to derive uptake parameters. Open symbols are experimentally determined values from fluorescent intensity, and closed symbols are predicted model outcomes.

Fig. S2. Calibration of tau QSP model uptake parameters to experimental data from internalization of brain extracts. The model is applied to tau-enriched brain extracts from AD and PSP patients in rat primary neuronal cultures to derive appropriate uptake parameters. AD-150: 150 ng tau; AD-450: 450 ng tau, CBD-150: 150 ng tau, PSP 1-1.50 ng tau, PSP 1-15: 15 ng tau, PSP 1-150: 150 ng tau, PSP 1-450: 450 ng tau [2]

Fig. S3. Calibration of the QSP model for a-synuclein oligomers. Uptake of fluorescently labelled a-synuclein oligomers in hIPSC cells is modelled to derive appropriate values. The X-axis is in nM, and the Y-axis is in A.U.

Fig. S4. PK profile of Gosuranemab. Different doses of gosuranemab from the observed Phase 1 study [4] are fitted to derive appropriate PK parameters. Symbols are experimentally determined plasma levels; full lines are predicted values. The model fit does not predict the first days very well, but the fit is significantly better for the later time points, as they drive the accumulated pharmacodynamic effect on tau dynamics.

Fig. S5. PK profile of Tilavonemab. Different doses of tilavonemab from the observed Phase 1 study [5] are fitted to derive appropriate PK parameters. Symbols are experimentally determined plasma levels; full lines are predicted values. The model fit does not predict the first days very well, but the fit is significantly better for the later time points, as they drive the accumulated pharmacodynamic effect on tau dynamics.

Fig. S6. PK profile of Semorinemab. Different doses of Semorinemab from the observed Phase 1 study [6] are fitted to derive appropriate PK parameters. Symbols are experimentally determined plasma levels; full lines are predicted values. The model fit does not predict the first days very well, but the fit is significantly better for the later time points, as they drive the accumulated pharmacodynamic effect on tau dynamics. Note that we additionally fitted a subcutaneous formulation of the same drug.

Fig. S7. PK profile of Prazineuzumab. Different doses of prazineuzumab from the observed Phase 1 study [7] are fitted to derive appropriate PK parameters. Symbols are experimentally determined plasma levels; full lines are predicted values. The model fit does not predict the first days very well, but the fit is significantly better for the later time points, as they drive the accumulated pharmacodynamic effect on tau dynamics.

Fig. S8. PK profile of Cinpanemab. Different doses of Cinpanemab from the observed Phase 1 study [8] are fitted to derive appropriate PK parameters PK profiles. Symbols are experimentally

determined plasma levels; full lines are predicted values. The model fit does not predict the first days very well, but the fit is significantly better for the later time points, as they drive the accumulated pharmacodynamic effect on tau dynamics.

Fig. S9. Gosanerumab in Alzheimer's disease. (a) Effect of gosuranemab on free CSF Tau. (a) in different compartments and synaptic cleft seed-competent tau (B) for a 52-week study at 60 mpk Q4 W in AD patients. The original pharmacology where the affinity for monomeric and seed-competent tau is identical (20 pM) leads to an almost full depletion of free CSF and ISF monomeric and seed-competent tau but almost no change in synaptic cleft monomeric and seed-competent tau. (b) The effect of gosuranemab on neuronal seed-competent au uptake is very limited (0.3% reduction at the highest dose).

Fig. S10. Changes in affinity of PFF Asyn to postsynaptic receptors. (a) Impact of affinity of PFF aSyn for postsynaptic receptors on PFF aSyn uptake reduction by cinpanemab 3500 mg, measured as the area under the curve (AUC) and normalized to the placebo case. The data show that increasing the affinity from the experimentally determined value of 800 nM leads to substantially reduced neuronal uptake with a steep drop for affinities higher than 10 nM. (b) Impact of the affinity of cinpanemab for seed-competent aSyn on the reduction in neuronal uptake. Lowering the affinity of the antibody for the seed-competent Asyn reduces the effect with a steep drop from 2 nM upwards.

Fig. S11. Change in affinity of oligomeric tau. (a) Impact on reduction in oligo tau neuronal uptake by gosuranemab 4500 mg as a function of the affinity of ***oligo tau*** for postsynaptic receptors, measured as the area under the curve (AUC) and normalized to the placebo case. The data show that decreasing the affinity from the experimentally determined value of 40 nM leads to a limited reduction in neuronal uptake. (b) Impact on reduction of oligo tau neuronal uptake

reduction by gosuranemab 4500 mg as a function of the affinity of ***monomeric tau*** for postsynaptic receptors. The data show that changing the affinity from the experimentally determined value of 800 nM does not affect the subsequent reduction in oligomeric tau uptake.

Table S1. Parameters of the model fitting. Experimental observations of in vitro datasets for uptake of different forms of seed-competent tau (brain extracts from AD, PSP and CBD) [2] and fluorescent tau oligomers [1].

Table S2. List of parameters after fitting the PK profiles. Koff-FcRn is the off-rate of the antibody from the FcR; sigma BCSFB is the dimensionless reflection coefficient at the Brain-CSF barrier, sigma_T_v is the tissue vascular reflection coefficient.

Table S3. Sensitivity analysis of changes in ISF to SynCleft flow. Impact of changes on the reduction of seed-competent tau and PFF aSyn uptake, measured as the area under the curve (AUC) and normalized to the placebo case. The simulations suggest that these changes do not substantially affect the pharmacodynamic effect of the antibodies on neuronal uptake.
